# Investigating cognitive flexibility and innovation in interdisciplinary project-based learning: the role of openness to learning and peer feedback quality in vocational education

**DOI:** 10.3389/fpsyg.2025.1691422

**Published:** 2025-11-20

**Authors:** Miao Xiong, Nee Nee Chan, Bee Eng Wong, Xiaomin Xie, Meng Na

**Affiliations:** 1 School of Mechanical Engineering, Chongqing Industry Polytechnic University, Chongqing, China; 2 Faculty of Social Sciences and Liberal Arts, UCSI University, Cheras, Malaysia; 3 Graduate School of Business, Universiti Kebangsaan Malaysia, Bangi, Selangor, Malaysia

**Keywords:** cognitive flexibility, interdisciplinary PBL, openness to learning, peer feedback quality, problem-solving, vocational education

## Abstract

This study investigates how cognitive flexibility, engagement, and teamwork interact to foster problem-solving and innovation within interdisciplinary project-based learning (PBL) in vocational education. Drawing on Cognitive Flexibility Theory (CFT), Social Interdependence Theory (SIT), and Transformative Learning Theory (TLT), it examines the contextual roles of project complexity and knowledge diversity, as well as the moderating effects of openness to learning and peer feedback quality. A cross-sectional survey of vocational students (*N* = 278) in Sichuan Province, China, was employed to test a structural model that assesses direct, mediated, and moderated relationships. Results indicate that project complexity and knowledge diversity significantly enhance cognitive flexibility, which in turn drives problem-solving and teamwork. Engagement further facilitates these outcomes, underscoring its role in collaborative and adaptive processes. Openness to learning and peer feedback quality exhibit nuanced moderating effects, highlighting that excessive openness or unstructured feedback can dilute rather than amplify innovation. The findings offer novel insights into how CFT, SIT, and TLT converge in interdisciplinary PBL, demonstrating that well-orchestrated team dynamics and contextual supports are essential for harnessing cognitive flexibility. Practically, the study provides actionable guidance for educators and policymakers seeking to design effective PBL environments, emphasizing the importance of structured peer feedback, balanced openness, and context-specific measurement of cognitive adaptability to meet the demands of contemporary workplaces.

## 1 Introduction

The ability to navigate complexity, adapt to novel situations, and collaborate effectively across disciplines is increasingly vital in today’s interconnected world. Vocational education, particularly through interdisciplinary project-based learning (PBL), provides a unique platform for cultivating these critical skills. Globally, approximately 75% of vocational training programs now include interdisciplinary components aimed at fostering adaptability and problem-solving capabilities (UNESCO, 2023). However, a pressing issue in vocational education is the misalignment between the demands of modern interdisciplinary challenges and students’ preparedness to adapt and innovate. In Sichuan Province, China, for example, with 2 million students enrolled in vocational training programs annually, a survey from the China Vocational Education Report (2024) found that 68% of these students faced difficulties in integrating knowledge across disciplines and collaborating effectively in team-based settings. These challenges are often illustrated when students from multiple departments—say, mechanical engineering, marketing, and computer science—struggle to synthesize their varied expertise in a single PBL project, resulting in fragmented efforts and suboptimal learning outcomes. Such scenarios underscore the need for clearer strategies to enhance cognitive flexibility and teamwork in interdisciplinary contexts.

Cognitive flexibility, defined as the ability to adapt thinking and behavior in response to changing contexts and feedback (Clément, 2022; Spiro et al., 1987), is central to addressing these challenges. Research suggests that enhancing cognitive flexibility can improve problem-solving efficiency by up to 35% in complex learning environments (Barrella et al., 2021). Yet, there is no consensus on how best to measure and foster cognitive flexibility in interdisciplinary PBL (Hidalgo and Ortega-Sánchez, 2022). For instance, one vocational college (?) piloted a cross-departmental design challenge where students from engineering and business had to collaborate on developing a sustainable product. Although the project yielded creative ideas, subsequent interviews revealed significant confusion over how to measure and track students’ growth in adaptability and integration of knowledge, highlighting the difficulty of operationalizing cognitive flexibility in real classroom settings.

Interdisciplinary PBL, characterized by collaborative problem-solving and knowledge integration, inherently promotes the development of cognitive flexibility (Clément, 2022; Liu et al., 2023). Students exposed to such learning environments have been shown to be 42% more likely to demonstrate improved cognitive adaptability compared to those in traditional, single-discipline programs (Zimmermann et al., 2010). However, the quality of peer feedback and the openness of learners to new ideas often determine whether these interdisciplinary projects fully realize their potential. For example, in a collaborative project involving health sciences and data analytics students, high-quality, constructive peer feedback helped some teams improve their project scope and enhance outcomes; yet, other teams encountered misaligned feedback or resistance to critique, limiting the project’s overall effectiveness. Such variations underscore the complexity of implementing peer feedback mechanisms and balancing them with learners’ receptiveness to alternative viewpoints (Hidalgo and Ortega-Sánchez, 2022; Yu, 2024).

Openness to learning is defined as a willingness to embrace new ideas and adapt to novel experiences, and this has been posited as a pivotal trait influencing cognitive flexibility (Chaijaroen, 2018). However, empirical examinations of how openness shapes interdisciplinary PBL are limited, often overlooking its potential to amplify or hinder group-level innovation and teamwork. In one instance, a vocational institute introduced a joint engineering-architecture course but found that students with lower openness to learning were more resistant to collaborating across disciplines, resulting in uneven team cohesion and stifled creativity (Nandan and London, 2013). Such examples reveal that a deeper understanding of how openness to learning moderates cognitive flexibility could inform targeted strategies to help learners better integrate knowledge and tackle complex, real-world issues.

To address these complexities, this study integrates Cognitive Flexibility Theory (CFT), Social Interdependence Theory (SIT), and Transformative Learning Theory (TLT), offering a multi-faceted lens to examine how cognitive adaptability, social mechanisms, and transformative processes converge in interdisciplinary PBL. CFT focuses on learners’ ability to restructure knowledge and adapt to complexity (Spiro et al., 1987). SIT highlights the role of team interdependence in shaping collective outcomes, including how feedback loops and collaboration drive problem-solving (Johnson and Johnson, 2008). Meanwhile, TLT introduces the importance of critical reflection and openness to learning in fostering innovation and adaptive change (Mezirow, 2018). By uniting these perspectives, this research aims to provide a robust framework for understanding and enhancing interdisciplinary PBL.

Ultimately, this study seeks to address the problem of suboptimal outcomes in interdisciplinary vocational education by examining how project complexity, knowledge diversity, engagement, and cognitive flexibility underpin problem-solving, teamwork, and innovation. It further explores how openness to learning and peer feedback quality moderate these relationships, offering a roadmap for educators, policymakers, and practitioners to design interventions that better equip learners with the adaptability, collaborative skills, and creative insight demanded by contemporary challenges. Through empirical evidence and illustrative examples, the study sheds light on the multifaceted nature of interdisciplinary PBL, paving the way for educational strategies that bridge theoretical ideals with the practical realities of vocational training contexts.

## 2 Literature review

### 2.1 Theoretical underpinning

Cognitive Flexibility Theory (CFT) emphasizes the importance of restructuring existing knowledge to adapt to novel or complex environments (Spiro et al., 1987). Traditionally, the theory is concerned with individual cognitive adaptability, focusing on internal schema restructuring to handle ill-structured problems. However, this study extends CFT to include external antecedents such as project complexity and knowledge diversity, which stimulate cognitive flexibility by exposing individuals to diverse, dynamic challenges. This extension aligns with evidence that complex tasks enhance cognitive adaptability and enable individuals to navigate uncertainty effectively (Spiro and Weitz, 1990). Furthermore, CFT’s scope is broadened to link cognitive flexibility not only to problem-solving but also to innovation, positioning it as a critical enabler in interdisciplinary team settings where adaptability drives creative outcomes. By situating cognitive flexibility within collaborative frameworks, the study demonstrates that this adaptability is not merely reactive but instrumental in shaping the dynamic interplay of team-based innovation.

Social Interdependence Theory (SIT) explains how positive interdependence among group members fosters cooperation, mutual accountability, and improved collective outcomes (Johnson and Johnson, 2008). While SIT traditionally focuses on group interdependence, this study integrates individual-level constructs like cognitive flexibility and openness to learning to understand how these factors interact with team dynamics. For instance, the role of peer feedback quality as a contextual moderator improves SIT by highlighting that while constructive feedback enhances team cohesion and innovation, excessive reliance on feedback may hinder decision-making processes, particularly in cognitively diverse teams (Hoegl and Parboteeah, 2003). This perspective expands SIT by clarifying the dual roles of feedback and interdependence in fostering or inhibiting collaboration and innovation. Additionally, the findings reveal that teamwork mediates the relationship between engagement, cognitive flexibility, and innovation, demonstrating that SIT can be enhanced by integrating mechanisms of cognitive adaptability (via CFT) and openness to learning.

Transformative Learning Theory (TLT) provides a new lens for examining the role of openness to learning in fostering adaptive and interdisciplinary skills within PBL. Mezirow (2018) argued that transformative learning involves critical reflection and the restructuring of perspectives, traditionally viewed as an individual process. However, this study situates TLT within a collaborative framework, illustrating how transformative learning emerges from collective engagement, teamwork, and cognitive adaptability. Openness to learning facilitates the integration of diverse perspectives, enabling individuals and teams to approach complex challenges with greater creativity and flexibility. The study also highlights that excessive openness may weaken the direct impact of engagement on performance outcomes, suggesting that TLT can benefit from a balanced approach that fosters adaptability while maintaining focus on goal alignment. By linking openness to learning with both cognitive flexibility and teamwork, the study expands TLT into domains where group-level transformation is essential for innovation.

**Figure 1:**
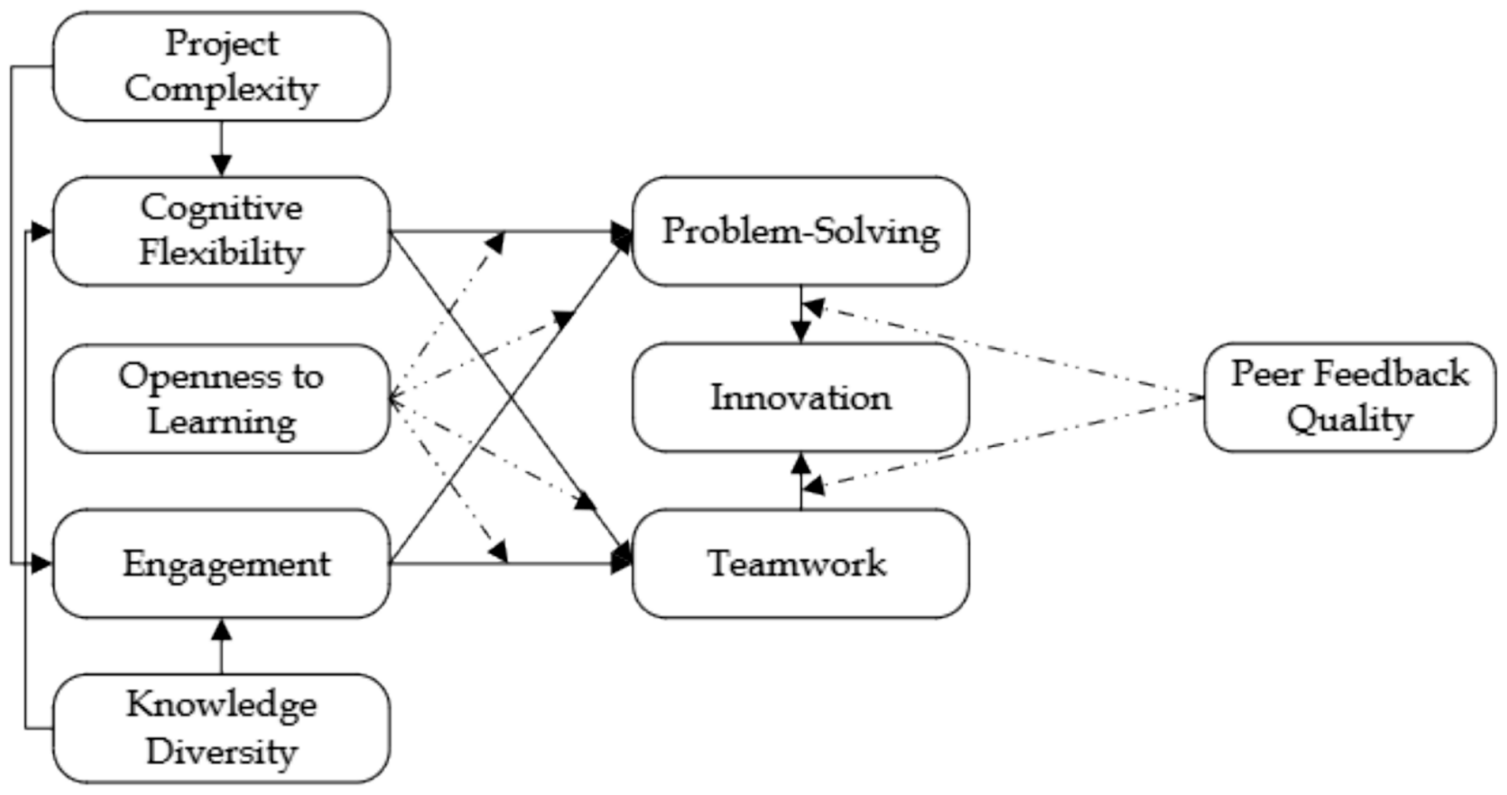
Research model.

While these three theories have distinct origins and emphases, their integration (Figure 1) in this study provides a cohesive framework for understanding interdisciplinary PBL. CFT focuses on the individual’s ability to adapt and restructure knowledge in complex contexts, SIT emphasizes the social mechanisms that foster collaboration and innovation, and TLT underscores the transformative potential of openness and critical reflection in learning. Together, these theories create a multidimensional lens that explains how cognitive, social, and transformative processes converge to enable collaborative problem-solving and innovation. This theoretical integration not only enriches the explanatory power of CFT, SIT, and TLT but also offers new pathways for exploring their application in dynamic, interdisciplinary educational and organizational contexts. By bridging individual and group dynamics, this framework provides a comprehensive understanding of how adaptability, interdependence, and transformative learning collectively drive innovation.

### 2.2 Project complexity and cognitive outcomes

Complex projects are characterized by high levels of uncertainty, interdependence, and the need for innovative solutions. These features inherently demand greater cognitive and emotional engagement from stakeholders (Doan and Trinh, 2024). Task complexity is a critical factor that predicts engagement, as challenging tasks stimulate motivation and active participation (Nguyen et al., 2021). Furthermore, cognitive flexibility—a mental skill that enables individuals to adapt to novel and dynamic contexts—becomes increasingly essential in navigating the intricacies of complex projects (Becker and Klarner, 2021). The reciprocal relationship between project complexity and cognitive flexibility underscores how engaging with multifaceted projects can both require and enhance flexible thinking (Kohn and Schooler, 1978). Research also highlights the role of early-stage project management practices, such as knowledge integration and interconnected learning strategies, in mitigating the adverse effects of complexity (Afshin et al., 2019). Therefore, it is posited that:

- *H1a*: Project complexity positively influences engagement.
- *H1b*: Project complexity positively influences cognitive flexibility.

### 2.3 Cognitive flexibility and performance outcomes

Cognitive flexibility is strongly linked to effective problem-solving, particularly in environments requiring innovative solutions. It allows individuals to approach problems from multiple perspectives, fostering adaptability and creativity (Idawati et al., 2020). This skill is critical not only for individual problem-solving but also for enhancing team dynamics and performance (Aggarwal et al., 2023). Teams with diverse cognitive resources benefit from increased creativity, conflict resolution, and social integration, leading to higher levels of collaboration and productivity (Jeffrey, 2003; Martins and Gonçalves, 2022). In team settings, cognitive flexibility facilitates the alignment of individual and collective goals, thereby enhancing teamwork efficiency (Shin et al., 2012). Accordingly:

- *H2a*: Cognitive flexibility positively influences problem-solving.
- *H2b*: Cognitive flexibility positively influences teamwork.

### 2.4 Engagement and performance outcomes

Engagement, defined as an individual’s emotional and cognitive investment in tasks, plays a pivotal role in achieving high problem-solving and teamwork outcomes (Lein et al., 2016). High engagement levels enhance focus and persistence, critical for solving complex problems (Jonathan et al., 2011). Furthermore, engagement fosters collaborative behaviors within teams by promoting shared perceptions and emotional alignment among members (Costa et al., 2014). Studies in gamified and narrative-centered learning environments demonstrate how engagement directly correlates with improved problem-solving skills and team cohesion (Pedro et al., 2012; Schöbel et al., 2019). Task conflict, when managed effectively, can strengthen these relationships, highlighting the dynamic role of engagement in complex team interactions (Costa et al., 2015). Therefore:

- *H3a*: Engagement positively influences problem-solving.
- *H3b*: Engagement positively influences teamwork.

### 2.5 Knowledge diversity, engagement, and cognitive flexibility

Knowledge diversity—the variety of perspectives, experiences, and expertise within a group—has been shown to positively influence engagement and cognitive flexibility across various contexts. In educational settings, mixed-knowledge groups demonstrate heightened behavioral, emotional, and social engagement compared to homogeneous knowledge groups (Zhao et al., 2018). Similarly, internal knowledge sharing enhances engagement and fosters collaborative climates in universities and organizations (Alshaabani et al., 2021; Selmer et al., 2012). Knowledge diversity facilitates cognitive flexibility by enabling individuals to draw upon diverse cognitive resources, leading to improved problem-solving and innovation (Paletz and Schunn, 2010; Sulik et al., 2021). Moreover, diversity in cognitive resources promotes openness to different perspectives, supporting decision-making and knowledge creation (Mitchell et al., 2009). These dynamics underscore the importance of knowledge diversity in stimulating both engagement and cognitive adaptability. Accordingly:

- *H4a*: Knowledge diversity positively influences engagement.
- *H4b*: Knowledge diversity positively influences cognitive flexibility.

### 2.6 Problem-solving as a mediator between engagement, cognitive flexibility, and innovation

Problem-solving serves as a critical mechanism linking engagement and cognitive flexibility to innovation. Engaged individuals exhibit enhanced problem-solving abilities, which contribute to technical and organizational innovations (Griffin and Guez, 2014; Jonathan et al., 2011). Cognitive flexibility, characterized by the ability to adapt thinking to novel and complex challenges, further enhances innovative outcomes by fostering active search and resource bricolage (Lyu et al., 2023). These capabilities allow individuals and teams to address uncertainty and generate creative solutions. Additionally, problem-solving skills mediate the impact of training and cognitive interventions on innovative thinking (Alescio-Lautier et al., 2021). Studies in team contexts highlight that collaborative problem-solving can transform diverse cognitive inputs into innovative outcomes, particularly in environments with moderate task conflict (De Dreu, 2006). Thus, problem-solving mediates the relationship between foundational constructs (engagement and cognitive flexibility) and innovation. Therefore:

- *H5a*: Problem-solving mediates the relationship between engagement and innovation.
- *H5b*: Problem-solving mediates the relationship between cognitive flexibility and innovation.

### 2.7 Teamwork as a mediator between engagement, cognitive flexibility, and innovation

Teamwork is a central factor in translating engagement and cognitive flexibility into innovative performance. Engaged teams exhibit higher levels of collaboration, communication, and adaptability, which are essential for fostering innovation (Ababneh, 2023; García-Buades et al., 2016). Cognitive diversity within teams enhances innovative behaviors through effective teamwork processes, such as knowledge sharing and constructive conflict management (Desivilya et al., 2010; Jankelová et al., 2021). Furthermore, team-level engagement mediates the relationship between leadership styles, such as transformational leadership, and open innovation (Edelbroek et al., 2019). Cognitive flexibility within teams allows members to navigate complex challenges and integrate diverse perspectives, enhancing team performance and innovative outcomes (Paletz and Schunn, 2010). These findings suggest that teamwork mediates the pathway from engagement and cognitive flexibility to innovation. Accordingly:

- *H6a*: Teamwork mediates the relationship between engagement and innovation.
- *H6b*: Teamwork mediates the relationship between cognitive flexibility and innovation.

### 2.8 Openness to learning, engagement, cognitive flexibility, and problem-solving

Openness to learning is a critical factor in enhancing engagement, cognitive flexibility, and problem-solving across educational and organizational settings. It fosters curiosity, adaptability, and willingness to embrace new ideas, leading to more effective learning and interpersonal interactions (Al-Muqaram and Al-Amara, 2017; Huang et al., 2023). Openness enhances the quality of engagement by encouraging learners to explore ideas and actively participate in collaborative tasks (Tjosvold et al., 2005). It also moderates the relationship between cognitive flexibility and problem-solving by promoting innovative thinking and persistence in overcoming challenges (Cui et al., 2023). Studies demonstrate that openness supports problem-solving in both structured and unstructured tasks, particularly when combined with external experiences, such as study abroad programs (Cho and Morris, 2015). Given its importance in facilitating learning and adaptive behaviors, openness to learning is expected to strengthen the influence of engagement and cognitive flexibility on problem-solving. Thus:

- *H7a*: Openness to learning moderates the influence of engagement on problem-solving.
- *H7b*: Openness to learning moderates the influence of cognitive flexibility on problem-solving.

### 2.9 Openness to learning, engagement, cognitive flexibility, and teamwork

In team-based environments, openness to learning plays a vital role in shaping engagement and cognitive flexibility, which are critical for effective teamwork. Teams with higher openness demonstrate improved decision-making, collaboration, and knowledge sharing (Colquitt et al., 2002; Cui et al., 2022). Openness fosters team learning and enhances the alignment of diverse perspectives, enabling members to work cohesively toward shared goals (Homan et al., 2008). Additionally, openness supports adaptive and reflective team behaviors, which are crucial in dynamic and cognitively diverse groups (Meslec and Graff, 2015). This adaptability further strengthens the relationship between engagement, cognitive flexibility, and teamwork outcomes, particularly in innovative and complex task settings (Mitchell et al., 2009). Therefore:

- *H8a*: Openness to learning moderates the influence of engagement on teamwork.
- *H8b*: Openness to learning moderates the influence of cognitive flexibility on teamwork.

### 2.10 Peer feedback quality, problem-solving, teamwork, and innovation

Peer feedback quality is a critical factor in enhancing problem-solving, teamwork, and innovation. Constructive and high-quality feedback fosters reflection, improves idea generation, and strengthens engagement within collaborative settings (Imam et al., 2024; Wang et al., 2023). Feedback mechanisms, such as task-specific comments and iterative reviews, contribute to improving problem-solving efficiency and innovative outputs (Seeber et al., 2017). In teamwork contexts, high-quality peer feedback promotes collaboration, knowledge sharing, and self-assessment abilities, thereby enhancing team performance (Díaz-Vicario et al., 2024). Furthermore, feedback quality moderates the relationship between teamwork and innovation by facilitating goal alignment and improving communication processes (Hoegl and Parboteeah, 2003). Given its role in fostering creativity and innovation, peer feedback quality is expected to amplify the influence of problem-solving and teamwork on innovative outcomes. Thus:

- *H9a*: Peer feedback quality moderates the influence of problem-solving on innovation.
- *H9b*: Peer feedback quality moderates the influence of teamwork on innovation.

## 3 Research methodology

### 3.1 Research design

This study employs a quantitative, cross-sectional design to investigate the relationships between cognitive flexibility, engagement, teamwork, peer feedback quality, and innovation in vocational training institutes in Sichuan Province, China. The target population comprises students enrolled in vocational training programs, emphasizing teamwork and project-based learning, making them ideal for the proposed constructs. A stratified random sampling approach was used across 12 vocational training institutions, ensuring balanced representation across institutions, disciplines, and regions, consistent with prior studies on diverse educational populations (Madaki et al., 2024).

The selection of these 12 institutions was based on predefined inclusion criteria: (1) offering interdisciplinary project-based learning (PBL) courses, (2) representing both public and private institutions, and (3) covering STEM (e.g., engineering) and non-STEM (e.g., business) disciplines to ensure knowledge diversity. Institutions that did not offer PBL-based programs or lacked interdisciplinary components in their curriculum were excluded from the sampling frame. The sample frame was constructed using the official registry of vocational training institutions from the Sichuan Province Education Bureau, categorizing institutions by ownership type, program offerings, and geographic location. Public and private institutions were included to capture governance diversity, while programs were stratified into STEM (e.g., engineering) and non-STEM (e.g., business) fields to reflect discipline-specific variations in teamwork and learning. Geographic stratification ensured representation of both urban and semi-urban regions, addressing regional disparities in educational practices (Ahmed et al., 2024). Within each stratum, students were randomly selected using class enrollment lists, ensuring proportional representation.

Using G*Power analysis, the required sample size was calculated based on a statistical power of 0.80, a medium effect size (*f*^2^ = 0.15), and a significance level of *α* = 0.05 (Cohen, 1988). A minimum of 200 respondents was determined, with a target of 300 respondents set to account for potential non-response and incomplete data (Hair et al., 2022). This additional 50% buffer ensures that the final usable dataset retains at least the required minimum of 200 valid responses after data cleaning. Previous studies have shown that response rates in survey-based research typically range from 60 to 75%, and setting a higher target helps mitigate missing data issues (Hair et al., 2022).

### 3.2 Instrumentation and translation process

A structured survey questionnaire was developed using validated scales to measure (Appendix) each construct of the study. The selection of items for each scale was guided by theoretical relevance, prior empirical validation, and psychometric reliability to ensure alignment with the study’s objectives. The number of items per construct was determined based on prior research recommendations, maintaining a balance between measurement precision and respondent burden.

- Engagement was assessed using 5 items adapted from the Utrecht Work Engagement Scale (UWES) (Schaufeli et al., 2006), focusing on vigor, dedication, and absorption to capture students’ active involvement in PBL settings.
- Cognitive flexibility was measured using 4 items adapted from Spiro et al. (1987), emphasizing adaptability and perspective-taking, as these dimensions are critical for interdisciplinary learning.
- Teamwork was evaluated using 3 items adapted from Jankelová et al. (2021), concentrating on collaboration, conflict resolution, and shared responsibility, as these factors directly influence group dynamics in PBL.
- Problem-solving was measured with 4 items adapted from Alescio-Lautier et al. (2021), covering strategy development, reflective improvement, and solution-oriented thinking, as these aspects are central to cognitive adaptability in learning environments.
- Openness to learning utilized 3 items derived from Cui et al. (2023), assessing willingness to embrace new ideas, adaptability, and receptiveness to feedback, which are key in fostering interdisciplinary innovation.
- Peer feedback quality was measured with 3 items adapted from Hattie and Timperley (2007), emphasizing constructiveness, timeliness, and applicability of feedback, as these aspects determine its effectiveness in enhancing learning.
- Innovation was assessed with 3 items from Griffin and Guez (2014), focusing on creativity, experimentation, and novel idea generation, as these attributes are key indicators of innovative behavior.
- Knowledge diversity was measured using 4 items adapted from Zhao et al. (2018), highlighting varied expertise and exposure to different disciplines, as diversity in knowledge sources strengthens interdisciplinary collaboration.
- Project complexity was evaluated using 4 items adapted from Nguyen et al. (2021), capturing task interdependence, uncertainty, and problem difficulty, which are crucial factors in assessing cognitive demands.

To ensure linguistic and conceptual equivalence, the questionnaire was translated into Mandarin using a back-translation method (Brislin, 1970). A pretest with 10 participants from the target population evaluated clarity, comprehension, and cultural relevance, with feedback incorporated following Hwang and Salvendy (2010) recommendations. A pilot study with 30 participants from two vocational training institutions tested the reliability and validity of the instrument, ensuring that the selected items effectively captured the diversity and contextual nuances of the main sample (Hair et al., 2022).

### 3.3 Data collection

Surveys were administered using a dual data collection strategy, online surveys were conducted via Qualtrics and Wenjuanxing, leveraging institutional networks for direct outreach to students through email and messaging platforms (e.g., WeChat). For participants with limited digital access or less likely to respond to online invitations, in-person surveys were conducted in semi-urban regions (refer to areas that exhibit characteristics of both urban and rural environments), ensuring inclusivity and equitable participation (Huang et al., 2023). Data collection was completed over 12 weeks (September–November 2024), yielding 278 valid responses (191 online, 87 in-person). The response rate was 62%, consistent with similar studies (Ahmed and Aziz, 2024). Data validation ensured minimal missing responses (<2%), addressed through follow-ups or imputation techniques.

### 3.4 Participants

The demographic profile (Table 1) of respondents provides critical context for understanding the dynamics of cognitive flexibility, engagement, teamwork, peer feedback quality, and innovation in vocational training students. The sample consists of 278 respondents, with a 54% males and 46% female and a predominant age group of 21–23 years (49.6%), reflecting the typical demographic of vocational students in Sichuan Province. Public institution students form the majority (63.3%), while 60.4% of respondents are enrolled in STEM fields, highlighting the emphasis on technical education in the region. Urban students (68.3%) are more represented than those from semi-urban areas (31.7%), reflecting the concentration of resources and institutions in cities.

**Table 1:** Demographics of the respondents.

Variable	Category	(*n*)	(%)
Gender	Male	150	54.0
	Female	128	46.0
Age group	18–20 years	112	40.3
	21–23 years	138	49.6
	24 years and above	28	10.1
Institution type	Public	176	63.3
	Private	102	36.7
Field of study	STEM	168	60.4
	Non-STEM	110	39.6
Region	Urban	190	68.3
	Semi-urban	88	31.7
Survey mode	Online	191	68.7
	In-person	87	31.3
Year of study	First year	98	35.3
	Second year	112	40.3
	Third year or above	68	24.4
Monthly household income	Below ¥3,000	92	33.1
	¥3,001–¥6,000	124	44.6
	Above ¥6,000	62	22.3
Work experience	No work experience	172	61.9
	Part-time work	94	33.8
	Full-time work	12	4.3
Primary motivation for enrolment	Career advancement	142	51.1
	Skill development	96	34.5
	Parental influence	40	14.4
Access to technology	Personal laptop/PC	208	74.8
	Shared devices	48	17.3
	No access	22	7.9
Frequency of team-based activities	Frequently	162	58.3
	Occasionally	96	34.5
	Rarely	20	7.2

The survey captured diverse perspectives through a mixed-mode administration, with 68.7% completing it online and 31.3% participating in person, ensuring inclusivity. Respondents spanned all years of study, with second-year students forming the largest group (40.3%). Socio-economic diversity is evident, with 44.6% of respondents from middle-income households and 33.1% from lower-income families. Most respondents (61.9%) have no prior work experience, while 33.8% have part-time experience, offering a mix of fresh and practical perspectives.

Motivations for enrollment vary, with career advancement being the most cited (51.1%), followed by skill development (34.5%) and parental influence (14.4%). Access to technology shows disparities: 74.8% own personal devices, but 17.3% rely on shared devices, and 7.9% lack access. Teamwork activities were a regular feature for most students, with 58.3% engaging frequently, aligning with the collaborative nature of vocational education. This demographic diversity ensures the findings are comprehensive and reflective of the targeted population’s educational and socio-economic contexts.

## 4 Results

### 4.1 Measurement model statistics

**Figure 2:**
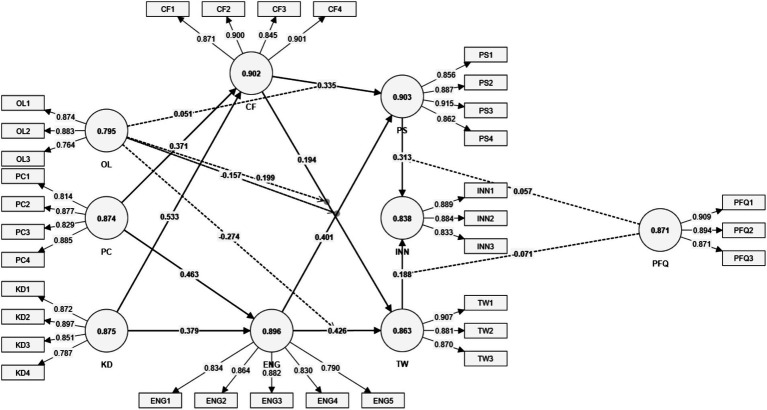
Measurement model (generated from data analysis with SmartPLS 4).

The evaluation of the measurement model (Figure 2) involved assessments of reliability, convergent validity, and discriminant validity (Table 2), ensuring robust construct measurement and alignment with established methodological standards. Outer loadings (OL) for all items were above the recommended threshold of 0.70, indicating strong individual item reliability, as per Hair et al. (2022).

**Table 2:** Measurement statistics.

Construct	Items	OL	VIF	CA	CR	AVE
CF	CF1	0.871	2.489	0.902	0.932	0.773
	CF2	0.900	2.934			
	CF3	0.845	2.262			
	CF4	0.901	3.052			
ENG	ENG1	0.834	2.420	0.896	0.923	0.707
	ENG2	0.864	2.544			
	ENG3	0.882	2.927			
	ENG4	0.830	2.100			
	ENG5	0.790	1.854			
INN	INN1	0.889	2.191	0.838	0.902	0.755
	INN2	0.884	2.126			
	INN3	0.833	1.740			
KD	KD1	0.872	2.532	0.875	0.914	0.727
	KD2	0.897	2.919			
	KD3	0.851	2.235			
	KD4	0.787	1.811			
OL	OL1	0.874	1.874	0.795	0.879	0.709
	OL2	0.883	1.951			
	OL3	0.764	1.473			
PC	PC1	0.814	1.931	0.874	0.914	0.726
	PC2	0.877	2.520			
	PC3	0.829	2.029			
	PC4	0.885	2.597			
PFQ	PFQ1	0.909	2.450	0.871	0.921	0.795
	PFQ2	0.894	2.390			
	PFQ3	0.871	2.148			
PS	PS1	0.856	2.332	0.903	0.932	0.775
	PS2	0.887	2.735			
	PS3	0.915	3.320			
	PS4	0.862	2.352			
TW	TW1	0.907	2.681	0.863	0.916	0.785
	TW2	0.881	2.069			
	TW3	0.870	2.179			

The loadings for cognitive flexibility (CF) ranged from 0.845 to 0.901, demonstrating high correlations between the construct and its items. These high loadings are essential for ensuring that the construct reliably measures the intended latent variable, reducing measurement errors (Hair et al., 2022).

Composite Reliability (CR) values, which ranged from 0.879 to 0.932 across constructs, exceeded the acceptable threshold of 0.70, confirming internal consistency (Hair et al., 2022). Similarly, Cronbach’s Alpha (CA) values for all constructs were above 0.70, with cognitive flexibility (CF) at 0.902 and Engagement (ENG) at 0.896, further establishing reliability. These values align with prior recommendations that emphasize high CR and CA as prerequisites for reliable scales in PLS-SEM models (Henseler et al., 2015). Convergent validity was also confirmed, as the Average Variance Extracted (AVE) for all constructs surpassed the 0.50 threshold, indicating that the constructs captured more than 50% of the variance in their respective items (Fornell and Larcker, 1981). For example, the AVE for Innovation (INN) was 0.755, affirming the construct’s ability to explain a substantial portion of its items’ variance.

Discriminant validity was evaluated using two complementary methods: the Heterotrait-Monotrait Ratio (HTMT; Table 3) and the Fornell-Larcker Criterion (FLC; Table 4). HTMT values for all construct pairs were below the threshold of 0.90, as advocated by Henseler et al. (2015), confirming that the constructs were sufficiently distinct from one another. For instance, the HTMT value between Cognitive Flexibility (CF) and Peer Feedback Quality (PFQ) was 0.804, while the value between Engagement (ENG) and Innovation (INN) was 0.814, both indicating strong discriminant validity. Similarly, the Fornell-Larcker Criterion verified that the square root of the AVE for each construct exceeded its correlations with other constructs. For example, the square root of the AVE for cognitive flexibility (CF) was 0.879, which was greater than its correlation with Engagement (0.717) and Peer Feedback Quality (0.783), further reinforcing discriminant validity (Fornell and Larcker, 1981).

**Table 3:** Discriminant validity (HTMT).

Construct	CF	ENG	INN	KD	OL	PC	PFQ	PS	TW	PFQ × TW	PFQ × PS	OL × CF	OL × ENG
CF													
ENG	0.794												
INN	0.826	0.814											
KD	0.844	0.788	0.816										
OL	0.458	0.493	0.494	0.454									
PC	0.836	0.818	0.843	0.793	0.382								
PFQ	0.804	0.837	0.723	0.873	0.499	0.838							
PS	0.782	0.819	0.836	0.787	0.500	0.775	0.827						
TW	0.640	0.744	0.802	0.652	0.447	0.753	0.768	0.827					
PFQ × TW	0.296	0.355	0.461	0.290	0.310	0.412	0.349	0.444	0.527				
PFQ × PS	0.386	0.362	0.471	0.364	0.350	0.383	0.429	0.554	0.430	0.809			
OL × CF	0.352	0.321	0.367	0.278	0.216	0.298	0.342	0.405	0.300	0.498	0.585		
OL × ENG	0.315	0.351	0.354	0.223	0.164	0.322	0.306	0.443	0.404	0.547	0.574	0.839	

**Table 4:** Discriminant validity (FLC).

Construct	CF	ENG	INN	KD	OL	PC	PFQ	PS	TW
CF	0.879								
ENG	0.717	0.841							
INN	0.764	0.775	0.869						
KD	0.792	0.702	0.763	0.853					
OL	0.391	0.419	0.405	0.381	0.842				
PC	0.743	0.727	0.742	0.698	0.320	0.852			
PFQ	0.783	0.745	0.836	0.766	0.414	0.735	0.892		
PS	0.709	0.740	0.808	0.707	0.425	0.690	0.737	0.880	
TW	0.571	0.662	0.769	0.575	0.377	0.658	0.673	0.765	0.886

Collinearity was assessed using the Variance Inflation Factor (VIF), with all values falling well below the critical threshold of 5.0, as recommended by Hair et al. (2022). VIF values ranged from 1.473 to 3.320, indicating no significant multicollinearity issues that could compromise the reliability of the constructs. For example, items under peer feedback quality (PFQ) showed VIF values between 2.148 and 2.450, reflecting a well-balanced and independent contribution of each item to the construct.

These robust results demonstrate that the measurement model satisfies key reliability and validity criteria. The constructs in the model—such as cognitive flexibility (CF), Engagement (ENG), and Innovation (INN)—are measured consistently and distinctly, providing confidence in the validity of the theoretical framework. The comprehensive approach to measurement model validation ensures that the constructs are well-positioned for the structural model analysis, as emphasized in contemporary guidelines for high-impact quantitative research.

### 4.2 Model fit and predictive relevance

The structural model’s fit and predictive relevance (Table 5) were evaluated using *R*^2^ (explanatory power), *R*^2^-adjusted (model stability), *Q*^2^-predict (predictive relevance), RMSE (Root Mean Square Error − prediction accuracy), and MAE (Mean Absolute Error − prediction error), aligning with PLS-SEM guidelines (Hair et al., 2011). The *R*^2^ values indicate strong explanatory power for most constructs, with Innovation (INN) having the highest *R*^2^ of 0.806, followed by cognitive flexibility (CF) at 0.697 and Problem-Solving (PS) at 0.649. Engagement (ENG) and Teamwork (TW) exhibit moderate variance explained, with *R*^2^ values of 0.603 and 0.510, respectively. The *R*^2^-adjusted values closely align with *R*^2^, confirming model stability and minimal overfitting.

**Table 5:** Model fit and predict.

Construct	*R* ^2^	*R*^2^ adjusted	*Q*^2^ predict	RMSE	MAE
CF	0.697	0.696	0.690	0.560	0.395
ENG	0.603	0.600	0.594	0.641	0.490
INN	0.806	0.803	0.744	0.510	0.393
PS	0.649	0.644	0.591	0.646	0.470
TW	0.510	0.503	0.462	0.743	0.560

The *Q*^2^-predict values, all above zero, demonstrate strong predictive relevance, particularly for INN (0.744), CF (0.690), and ENG (0.594). RMSE and MAE values indicate high accuracy, with the lowest errors observed for INN (RMSE: 0.510, MAE: 0.393) and CF (RMSE: 0.560, MAE: 0.395). These metrics validate the model’s precision and predictive reliability.

Overall, the model exhibits robust explanatory power and predictive relevance, effectively capturing the relationships among cognitive flexibility, engagement, teamwork, and innovation, meeting advanced evaluation standards for PLS-SEM models (Hair et al., 2022).

### 4.3 Hypothesis testing and discussion

**Figure 3:**
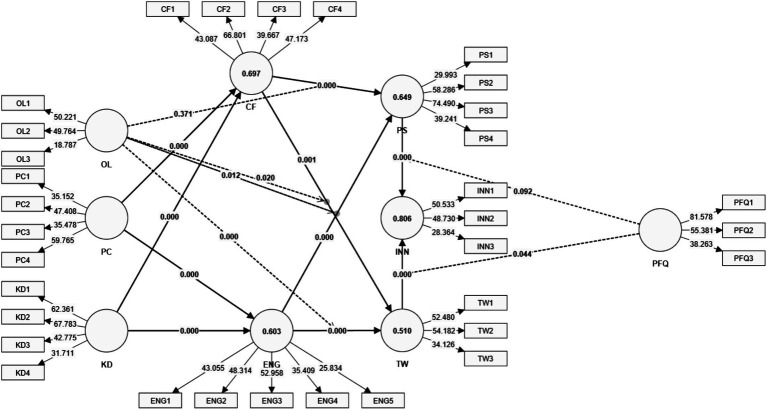
Structural model.

The structural model (Figure 3) analysis provides in-depth insights into the hypothesized relationships, with most supported by significant path coefficients (*p* < 0.05) and substantial effect sizes (*f*^2^; Table 6), validating the robustness of the theoretical framework. The results affirm the importance of project complexity, cognitive flexibility, engagement, and knowledge diversity as pivotal drivers of problem-solving, teamwork, and innovation in vocational education.

**Table 6:** Structural model statistics.

Hypothesis path		Original sample	Sample mean	Standard deviation	*T* statistics	*p* values	*f* ^2^	Support
*H1a*	PC → ENG	0.463	0.464	0.062	7.529	0.000	0.277	Yes
*H1b*	PC → CF	0.371	0.371	0.057	6.460	0.000	0.234	Yes
*H2a*	CF → PS	0.335	0.340	0.064	5.217	0.000	0.147	Yes
*H2b*	CF → TW	0.194	0.200	0.061	3.204	0.001	0.035	Yes
*H3a*	ENG → PS	0.401	0.400	0.059	6.847	0.000	0.203	Yes
*H3b*	ENG → TW	0.426	0.423	0.063	6.743	0.000	0.165	Yes
*H4a*	KD → ENG	0.379	0.378	0.066	5.738	0.000	0.185	Yes
*H4b*	KD → CF	0.533	0.533	0.058	9.217	0.000	0.482	Yes
*H5a*	ENG → PS → INN	0.125	0.126	0.030	4.227	0.000		Yes
*H5b*	OL → PS → INN	0.033	0.033	0.015	2.239	0.025		Yes
*H6a*	ENG → TW → INN	0.080	0.080	0.028	2.889	0.004		Yes
*H6b*	CF → TW → INN	0.036	0.037	0.015	2.399	0.016		Yes
*H7a*	OL × ENG → PS	−0.157	−0.153	0.062	2.513	0.012	0.039	Yes
*H7b*	OL × CF → PS	0.051	0.048	0.057	0.895	0.371	0.004	No
*H8a*	OL × ENG → TW	−0.274	−0.269	0.074	3.689	0.000	0.085	Yes
*H8b*	OL × CF → TW	0.199	0.196	0.085	2.332	0.020	0.043	Yes
*H9a*	PFQ × PS → INN	0.057	0.054	0.034	1.683	0.092	0.014	No
*H9b*	PFQ × TW → INN	−0.071	−0.067	0.035	2.018	0.044	0.021	Yes

For *H1*, Project Complexity (PC) significantly predicts Engagement (ENG) (*H1a*, *β* = 0.463, *t* = 7.529, *p* = 0.000) and cognitive flexibility (CF) (*H1b*, *β* = 0.371, *t* = 6.460, *p* = 0.000). These findings are consistent with prior studies that highlight the role of complex tasks in fostering emotional and cognitive involvement (Doan and Trinh, 2024). Complex projects act as motivators, encouraging active participation and adaptability, particularly in environments that emphasize teamwork and problem-solving (Nguyen et al., 2021). The reciprocal relationship between complexity and flexibility reflects that not only do complex projects demand cognitive adaptability, but they also cultivate it over time (Kohn and Schooler, 1978). Moderate effect sizes (*f*^2^ = 0.277 for ENG, *f*^2^ = 0.234 for CF) support the substantial influence of PC, further corroborated by literature on interconnected learning strategies as a response to complexity (Afshin et al., 2019).

For H2, cognitive flexibility (CF) significantly enhances problem-solving (PS) (*H2a*, *β* = 0.335, *t* = 5.217, *p* = 0.000) and Teamwork (TW) (*H2b*, *β* = 0.194, *t* = 3.204, *p* = 0.001). CF facilitates the use of diverse problem-solving approaches, critical for managing novel challenges, and fosters team collaboration by enabling alignment among group members (Aggarwal et al., 2023; Idawati et al., 2020). While the effect size for PS (*f*^2^ = 0.147) emphasizes its critical role in individual and group performance, the smaller, though significant, effect size for TW (*f*^2^ = 0.035) highlights CF’s role in promoting social integration and resolving conflicts within teams (Martins and Gonçalves, 2022; Shin et al., 2012).

*H3* findings reveal that Engagement (ENG) significantly predicts PS (*H3a*, *β* = 0.401, *t* = 6.847, *p* = 0.000) and TW (*H3b*, *β* = 0.426, *t* = 6.743, *p* = 0.000), underscoring the importance of emotional and cognitive investment in task performance. ENG enhances focus and persistence, traits critical for solving complex problems (Lein et al., 2016), and fosters collaborative behaviors, promoting shared perceptions and emotional alignment among team members (Costa et al., 2014; Costa et al., 2015). Substantial effect sizes (*f*^2^ = 0.203 for PS, *f*^2^ = 0.165 for TW) highlight the role of engagement in driving both individual and team-level outcomes, consistent with evidence from gamified and narrative-centered learning environments (Pedro et al., 2012; Schöbel et al., 2019).

*H4* demonstrates that Knowledge Diversity (KD) significantly influences ENG (*H4a*, *β* = 0.379, *t* = 5.738, *p* = 0.000) and CF (*H4b*, *β* = 0.533, *t* = 9.217, *p* = 0.000), with a particularly strong effect on CF (*f*^2^ = 0.482). KD enriches cognitive adaptability and engagement by exposing individuals to varied perspectives, fostering openness and sustained participation (Paletz and Schunn, 2010; Zhao et al., 2018). This finding underscores the importance of diverse cognitive resources in facilitating adaptability and decision-making (Mitchell et al., 2009; Selmer et al., 2012).

For *H5* and *H6*, the mediating roles of PS and TW highlight their importance in linking ENG and CF to Innovation (INN). ENG influences INN through PS (*β* = 0.125, *t* = 4.227, *p* = 0.000) and TW (*β* = 0.080, *t* = 2.889, *p* = 0.004), while CF affects INN via TW (*β* = 0.036, *t* = 2.399, *p* = 0.016). These results highlight the pathways through which foundational constructs like engagement and flexibility are translated into innovative outcomes. Collaborative problem-solving plays a crucial role, allowing engaged individuals to generate creative solutions and address uncertainty effectively (Griffin and Guez, 2014; Lyu et al., 2023). Similarly, teamwork processes leverage cognitive diversity, enabling teams to integrate diverse inputs into novel outputs (De Dreu, 2006; García-Buades et al., 2016).

The moderating effects of openness to learning (OL) reveal nuanced dynamics. OL negatively moderates the ENG-PS relationship (*β* = −0.157, *t* = 2.513, *p* = 0.012), suggesting that excessive openness may dilute engagement’s direct impact on problem-solving due to cognitive overload or decision fatigue (Huang et al., 2023). Conversely, OL strengthens the CF-TW relationship (*β* = 0.199, *t* = 2.332, *p* = 0.020), supporting adaptability and perspective alignment in teams (Cui et al., 2022). The negative moderation of ENG-TW (*β* = −0.274, *t* = 3.689, *p* = 0.000) reflects potential disruptions in collaboration when openness introduces conflicting viewpoints or challenges in achieving shared goals (Homan et al., 2008).

For peer feedback quality (PFQ), mixed results were observed. While PFQ does not significantly moderate the PS-INN relationship (*β* = 0.057, *t* = 1.683, *p* = 0.092), it negatively moderates TW-INN (*β* = −0.071, *t* = 2.018, *p* = 0.044), indicating that overreliance on feedback may hinder teamwork cohesion and delay decision-making processes (Díaz-Vicario et al., 2024).

In conclusion, the findings provide substantial evidence supporting the theoretical framework, demonstrating the critical roles of PC, KD, ENG, and CF in driving PS, TW, and INN. The mediating roles of PS and TW elucidate the mechanisms through which foundational constructs impact innovation, while the moderating effects of OL and PFQ underscore the contextual factors that shape these dynamics. These results align with and extend existing literature, offering nuanced insights into the interplay of individual and team-level traits in fostering adaptability and innovation in educational and vocational settings.

## 5 Implications

### 5.1 Theoretical implications

This study provides noteworthy contributions to Cognitive Flexibility Theory (CFT), Social Interdependence Theory (SIT), and Transformative Learning Theory (TLT) by situating them within the context of vocational education and innovation. It broadens CFT beyond individual learning mechanisms by demonstrating how project complexity and knowledge diversity serve as antecedents that drive cognitive flexibility. Traditionally, CFT focuses on internal adaptations and schema restructuring, but these findings integrate external and collaborative dimensions, illustrating how environmental complexity and diverse perspectives enhance the ability to respond to and create complex problem-solving contexts. The identification of innovation as an outcome of adaptive thinking further extends CFT by linking cognitive flexibility to creative and interdisciplinary endeavors. In advancing SIT, this research highlights the interplay between teamwork, engagement, and openness to learning in fostering collective innovation. While SIT traditionally emphasizes positive interdependence, the results underscore that constructs such as cognitive flexibility and peer feedback quality can enhance or disrupt collaborative processes, offering a more context-sensitive interpretation of how team interdependence influences group performance.

Additionally, the study enriches TLT by emphasizing that transformative learning can emerge from collective engagement, problem-solving, and cognitive adaptability, not solely from individual reflection. The demonstration that teamwork and openness to learning catalyze transformative experiences at both the individual and group levels bridges the gap between personal transformation and collaborative innovation, thereby positioning TLT within a broader, team-based framework.

### 5.2 Practical implications

The findings carry practical significance for educational institutions, vocational training centers, and policymakers seeking to cultivate collaborative problem-solving and innovation. By confirming the importance of project complexity, knowledge diversity, and engagement, the study suggests that curricula should integrate real-world, interdisciplinary projects requiring learners to navigate uncertainty and interdependence. This approach not only encourages active participation and cognitive flexibility but also prepares students for the dynamic nature of modern workplaces. Institutions can promote knowledge diversity by assembling teams with varied disciplinary backgrounds and adopting enrollment policies that encourage heterogeneity. Such diversity, when paired with reflective practices and adaptive learning technologies, strengthens learners’ capacity to meet new challenges and adapt to diverse perspectives.

Engagement emerges as a key driver for both individual and team-level outcomes, underscoring the need for instructional strategies that spark intrinsic motivation and teamwork, such as gamified tasks and narrative-centered projects. Yet, the role of openness to learning in moderating these relationships indicates that educators must strike a balance between encouraging exploratory thinking and providing sufficient structure to prevent cognitive overload or misalignment. Integrating constructive, high-quality peer feedback is equally crucial for refining ideas and enhancing team cohesion, although excessive reliance on iterative reviews may hinder decision-making.

Finally, policymakers should adopt systemic measures that support interdisciplinary collaboration, experiential learning, and collaborations with industry partners, thereby ensuring that educators receive the training and resources necessary to guide learners toward innovation.

### 5.3 Conclusion and future research directions

This study underscores the interdependence of cognitive flexibility, engagement, teamwork, and peer feedback quality in fostering innovation within vocational education settings. It validates the proposed theoretical framework by demonstrating that project complexity and knowledge diversity significantly drive engagement and cognitive flexibility, which in turn enable problem-solving, teamwork, and innovation. The mediating roles of problem-solving and teamwork clarify how core attributes translate into innovative performance, while the moderating effects of openness to learning and peer feedback quality reveal contextual conditions that strengthen or constrain collaborative outcomes.

These findings offer practical implications for curriculum design, instructional strategies, and institutional policymaking in vocational education. However, they also open multiple avenues for future research. First, comparative studies in different cultural and organizational contexts could enhance generalizability. Second, longitudinal and experimental designs are recommended to better establish causal linkages between project complexity, knowledge diversity, engagement, cognitive flexibility, and innovation. Third, future inquiries may integrate emerging technologies such as artificial intelligence and adaptive learning platforms to examine new mechanisms for enhancing teamwork and creativity. Finally, extending investigations into corporate or non-academic settings would bridge educational frameworks with real-world innovation practices. Collectively, this study provides a foundation for continued exploration of how cognitive, social, and contextual elements interact to cultivate creativity and adaptability in both educational and professional domains.

### 5.4 Limitations of the study

Despite its contributions, several limitations should be acknowledged. First, the cross-sectional design constrains causal inference; future work should employ longitudinal tracking or experimental interventions to capture dynamic changes in cognitive flexibility, engagement, and teamwork over time. Second, the study was conducted within vocational education institutions in Sichuan Province, China, which may limit transferability to other cultural or educational contexts. Third, reliance on self-reported survey data introduces potential response bias; incorporating objective performance indicators, peer assessments, or behavioral analytics could strengthen measurement validity. Fourth, while openness to learning and peer feedback quality were tested as moderators, additional contextual factors—such as leadership style, institutional culture, or digital learning tools—may further shape interdisciplinary PBL effectiveness. Finally, the growing presence of AI-driven interventions suggests an opportunity for future research to examine how algorithmic feedback, adaptive systems, or collaborative technologies can enhance cognitive flexibility and innovation in team-based learning environments.

## Data Availability

The raw data supporting the conclusions of this article will be made available by the authors, without undue reservation.

## Appendix

**Table A1:** Measurement items

Construct	Item	Source
Cognitive flexibility (CF)	CF1: I can easily adjust my thinking when faced with new information.	Spiro et al. (1987)
	CF2: I can consider multiple perspectives when solving a problem.	
	CF3: I adapt my approach to meet the demands of different situations.	
	CF4: I can integrate diverse knowledge to address complex challenges.	
Engagement (ENG)	ENG1: I feel enthusiastic about the tasks assigned in interdisciplinary projects.	Schaufeli et al. (2006)
	ENG2: I invest significant energy into completing project-related tasks.	
	ENG3: I stay focused and committed when working on team-based projects.	
	ENG4: I actively participate in discussions and contribute ideas in team settings.	
	ENG5: I find the tasks in interdisciplinary projects mentally stimulating.	
Innovation (INN)	INN1: I frequently generate creative ideas to address project challenges.	Griffin and Guez (2014)
	INN2: I propose new approaches to improve project outcomes.	
	INN3: I actively experiment with innovative solutions during project tasks.	
Knowledge diversity (KD)	KD1: My team comprises members with varied expertise and skills.	Zhao et al. (2018)
	KD2: I often collaborate with individuals from diverse educational backgrounds.	
	KD3: Working with team members from different disciplines enhances our project outcomes.	
	KD4: Knowledge diversity in my team encourages innovative solutions.	
Openness to learning (OL)	OL1: I am eager to explore new ideas during project discussions.	Cui et al. (2023)
	OL2: I willingly accept feedback that challenges my existing beliefs.	
	OL3: I adapt my views when exposed to new and convincing evidence.	
Project complexity (PC)	PC1: The projects I work on involve solving complex and interconnected problems.	Nguyen et al. (2021)
	PC2: My projects require integrating knowledge from multiple disciplines.	
	PC3: The tasks in my projects demand significant problem-solving skills.	
	PC4: Managing dependencies and constraints in my projects is challenging.	
Peer feedback quality (PFQ)	PFQ1: The feedback I receive from peers is constructive and actionable.	Hattie and Timperley (2007)
	PFQ2: My peers provide feedback that enhances the quality of my work.	
	PFQ3: Peer feedback in my projects is clear, specific, and timely.	
Problem-solving (PS)	PS1: I can identify effective strategies to address project challenges.	Alescio-Lautier et al. (2021)
	PS2: I systematically evaluate potential solutions to problems.	
	PS3: I successfully implement solutions to achieve project goals.	
	PS4: I reflect on past experiences to improve problem-solving in future tasks.	
Teamwork (TW)	TW1: My team collaborates effectively to achieve common project objectives.	Jankelová et al. (2021)
	TW2: Team members actively share their knowledge and expertise.	
	TW3: My team resolves conflicts constructively to ensure project success.	
